# Clinical-grade endometrial cancer detection system *via* whole-slide images using deep learning

**DOI:** 10.3389/fonc.2022.1040238

**Published:** 2022-11-02

**Authors:** Xiaobo Zhang, Wei Ba, Xiaoya Zhao, Chen Wang, Qiting Li, Yinli Zhang, Shanshan Lu, Lang Wang, Shuhao Wang, Zhigang Song, Danhua Shen

**Affiliations:** ^1^ Department of Pathology, Peking University People’s Hospital, Beijing, China; ^2^ Department of Pathology, Chinese PLA General Hospital, Beijing, China; ^3^ R&D Department, China Academy of Launch Vehicle Technology, Beijing, China; ^4^ Thorough Lab, Thorough Future, Beijing, China

**Keywords:** endometrial cancer, deep learning, artificial intelligence, convolutional neural network, data analysis

## Abstract

The accurate pathological diagnosis of endometrial cancer (EC) improves the curative effect and reduces the mortality rate. Deep learning has demonstrated expert-level performance in pathological diagnosis of a variety of organ systems using whole-slide images (WSIs). It is urgent to build the deep learning system for endometrial cancer detection using WSIs. The deep learning model was trained and validated using a dataset of 601 WSIs from PUPH. The model performance was tested on three independent datasets containing a total of 1,190 WSIs. For the retrospective test, we evaluated the model performance on 581 WSIs from PUPH. In the prospective study, 317 consecutive WSIs from PUPH were collected from April 2022 to May 2022. To further evaluate the generalizability of the model, 292 WSIs were gathered from PLAHG as part of the external test set. The predictions were thoroughly analyzed by expert pathologists. The model achieved an area under the receiver operating characteristic curve (AUC), sensitivity, and specificity of 0.928, 0.924, and 0.801, respectively, on 1,190 WSIs in classifying EC and non-EC. On the retrospective dataset from PUPH/PLAGH, the model achieved an AUC, sensitivity, and specificity of 0.948/0.971, 0.928/0.947, and 0.80/0.938, respectively. On the prospective dataset, the AUC, sensitivity, and specificity were, in order, 0.933, 0.934, and 0.837. Falsely predicted results were analyzed to further improve the pathologists’ confidence in the model. The deep learning model achieved a high degree of accuracy in identifying EC using WSIs. By pre-screening the suspicious EC regions, it would serve as an assisted diagnostic tool to improve working efficiency for pathologists.

## Introduction

Endometrial cancer (EC) is one of the most common gynecological tumors in women, with increasing incidence and mortality rates across the world (1, 2). In developed countries, EC ranks first in malignancies of the female reproductive system (2, 3). In the United States, it was estimated that 52,600 new cases of EC were reported in 2014, which increased to 61,880 in 2019, and the incidence continues to increase (2, 4, 5). According to the statistics of the National Cancer Center of China in 2019, the incidence of EC was 10.28/100,000, while the death rate was 1.9/100,000 (6, 7). In recent years, due to high-fat, high-fever diet, and low-exercise lifestyles, the incidence rate of EC in China has been rising (3, 7).

In clinical work, pathological diagnosis is the gold standard for endometrial specimens. Only when pathologists made accurate diagnoses, gynecologists would give next-step treatment suggestions, leading to an excessive demand for pathologists. The shortage of anatomic pathologists happens both in China and globally, resulting in an overloading of the workforce, thus affecting the diagnostic accuracy (8). According to the China Diagnostic Pathology Industry Analysis Report, the country requires 84,000–168,000 pathologists based on the need for 1-2 pathologists per 100 beds. However, as of 2018, there are only 18,000 pathologists on record, leaving a gap of at least 66,000 pathologists.

The majority of ECs can be diagnosed with curettage or biopsies, after which further surgical treatment is administered. Despite the huge volume of daily diagnostic requirements, specimens obtained during curettage or biopsies are frequently fragmented and asymmetrical, containing blood and even cervical mucus, which increases diagnostic complexity. These circumstances exert considerable pressure on the pathological diagnosis. The complexity of diagnosis and the lack of pathologists constitute a significant contradiction. It is worthwhile to investigate how to find new technologies that enable pathologists to concentrate on regions of interest (ROIs).

In recent years, artificial intelligence has seen tremendous growth, and the application of this cutting-edge technology to the area of pathology has gradually become a new trend. The latest studies have demonstrated that deep learning can be applied in the pathological diagnosis of a variety of organs, such as the prostate (9, 10), stomach (11–13), melanoma (14), lymph node metastasis (15), etc. In these studies, deep learning models can be used as a screening tool to flag the suspected malignant area in advance, prompting pathologists to thoroughly examine the ROIs, thus improving the diagnostic accuracy and shortening diagnostic time. There is also several research on the application of deep learning to EC recognition using whole-slide images (WSIs) (16–18). Sun et al. developed a convolutional neural network (CNN) to interpret hematoxylin and eosin (H&E)-stained image patches from endometrial specimens (16). This study classified pathological images at the patch level and used retrospectively collected cases for model evaluation. Zhao et al. developed a CNN to screen for endometrial intraepithelial neoplasia (17). Hong et al. trained a CNN to predict EC subtypes and molecular features (18).

In this research, we established a high-accuracy deep learning model for EC detection and conducted retrospective, prospective, and multicenter studies to demonstrate its clinical utility. The deep learning model achieved high sensitivity in detecting EC using WSIs with an area under the receiver operating characteristic (ROC) curve (AUC), sensitivity, and specificity of 0.928, 0.924, and 0.801, respectively, on 1,190 WSIs collected from the Peking University People’s Hospital (PUPH) and the Chinese PLA General Hospital (PLAGH). By studying the model predictions, we found the deep learning model was able to detect ECs with different morphology types, especially for illusory appearances.

## Materials and methods

### Dataset

To train and validate the CNN for EC detection, a total of 601 (551 for training and 50 for validation) slides of endometrial specimens from 601 patients were collected from PUPH, including all main pathological subtypes of the endometrium (**Table 1**). The cases of secretory phase, proliferative phase, endometrial hyperplasia without atypia, and endometrial atypical hyperplasia/endometrioid intraepithelial neoplasia were considered non-cancer. According to the World Health Organization classification of the female genitals, low-grade and high-grade ECs correspond to grades 1 & 2 and 3, respectively (19, 20). All slides were reviewed by two senior gynecological pathologists to reach an agreement on the final diagnostic report.

**Table 1:** Characteristics of the whole-slide images of endometrial specimens.

Dataset	Histological subtype	Training set	Validation set	Test set (PUPH, retrospective)	Test set (PLAGH, retrospective)	Test set (PUPH, prospective)
**Non-EC**	Secretory phase	88	12	86	19	67
	Proliferative phase	54	3	69	34	114
	Endometrial hyperplasia without atypia	40	5	17	8	23
	Endometrial atypical hyperplasia/ Endometrioid intraepithelial neoplasia	23	4	20	4	17
**EC**	EC (low-grade)	291	23	44	179	96
	EC (high-grade)	55	3	345	48	0
**Total**	1,190	551	50	581	292	317

EC, Endometrial carcinoma; PUPH, Peking University People’s Hospital; PLAGH, Chinese PLA General Hospital; WSIs, Whole-slide images.

The test set could be divided into retrospective and prospective parts. For the retrospective study, we collected 581 cases of endometrial biopsy specimens from PUPH and 292 cases from PLAGH. Meanwhile, 317 consecutive endometrial biopsies from PUPH were collected from April 2022 to May 2022 as the prospective test set. The gold-standard diagnoses of all cases in the test set were determined by two experts (X. Zhang and Z. Song) under a multi-head microscope. For cases with different diagnoses, consensus was reached after discussion.

All training and validation slides were digitized (eyepiece magnification fixed at 10x) using the KF-PRO-005 scanner (KFBio). The test slides from PUPH were digitalized using both KF-PRO-005 and Motic EasyScan 102. The test slides from PLAGH were scanned by Motic EasyScan 102. All the WSIs had a maximum zoom ratio of 400x and a physical resolution of 0.238μm/pixel.

### Data annotation

Expert pathologists labeled 346 training and 26 validation slides containing EC using a self-developed annotation system based on iPad, referring to the Fourth Edition of the WHO Classification of Tumors of the Endometrial System. A three-step approach including initial labeling, further verification, and final expert review (by D. Shen) was developed. Once the labeling was completed, the slides and annotations were sent to the training process.

### Model development

Using Otsu’s method, the background parts of the slide were filtered away while producing training and validation sets. Otsu’s method is one of the foreground detection approaches in computer vision. On the thumbnail of the grayscale slide, a grid search of the thresholding parameter was done to reduce the intra-class variance. This led to the extraction of the effective tissue area. The slides were then divided into patches of 320×320 pixels. These patches were extracted side-by-side from the effective tissue area without overlapping. Explicitly, 891,330 malignant and 1,983,966 benign patches were used for training.

**Figure 1:**
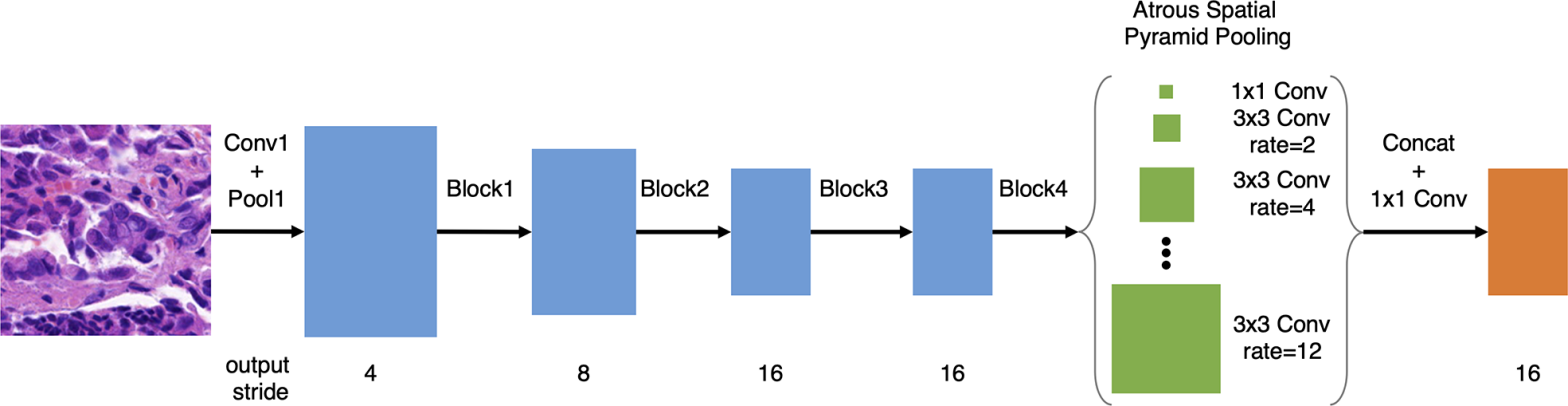
Deep learning model architecture.

Following our previous work on gastric cancer detection (13), based on DeepLab v3 and ResNet-50, we improved the model with atrous spatial pyramid pooling (ASPP) with dilations of 2, 4, 6, 8, 10, and 12, as illustrated in **Figure 1**, which improved the multi-scale detection capability. Meanwhile, we removed the image-level pooling information from the ASPP to force the model to focus on tumor cells. Since histology slides have no discernible orientation, we augmented the training data using random rotation and mirroring. We used random scaling between 1.0x and 1.5x to make the deep learning model more tolerant of slight variations in the scanning ratio. We further randomized the magnitude of the patch brightness, contrast, hue (average color), and saturation (with a maximum delta of 0.08).

**Figure 2:**
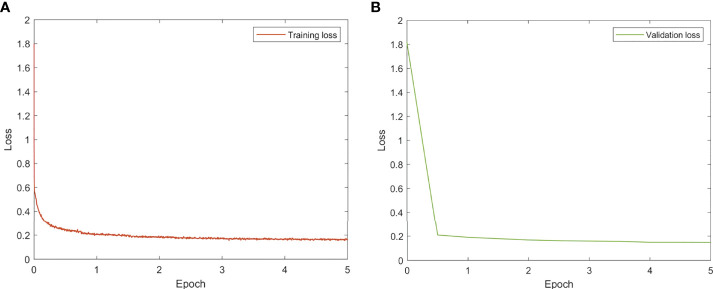
Learning curves of the deep learning model. **(A)** Training loss. **(B)** Validation loss.

All models were trained and evaluated on an Ubuntu server with four Nvidia GTX1080Ti GPUs using TensorFlow. To train the models, the ADAM optimizer with a fixed learning rate of 0.001 was utilized. The batch size was set at 80 (20 per GPU) and training was terminated after 5 epochs. The learning curves are given in **Figure 2**.

**Figure 3:**
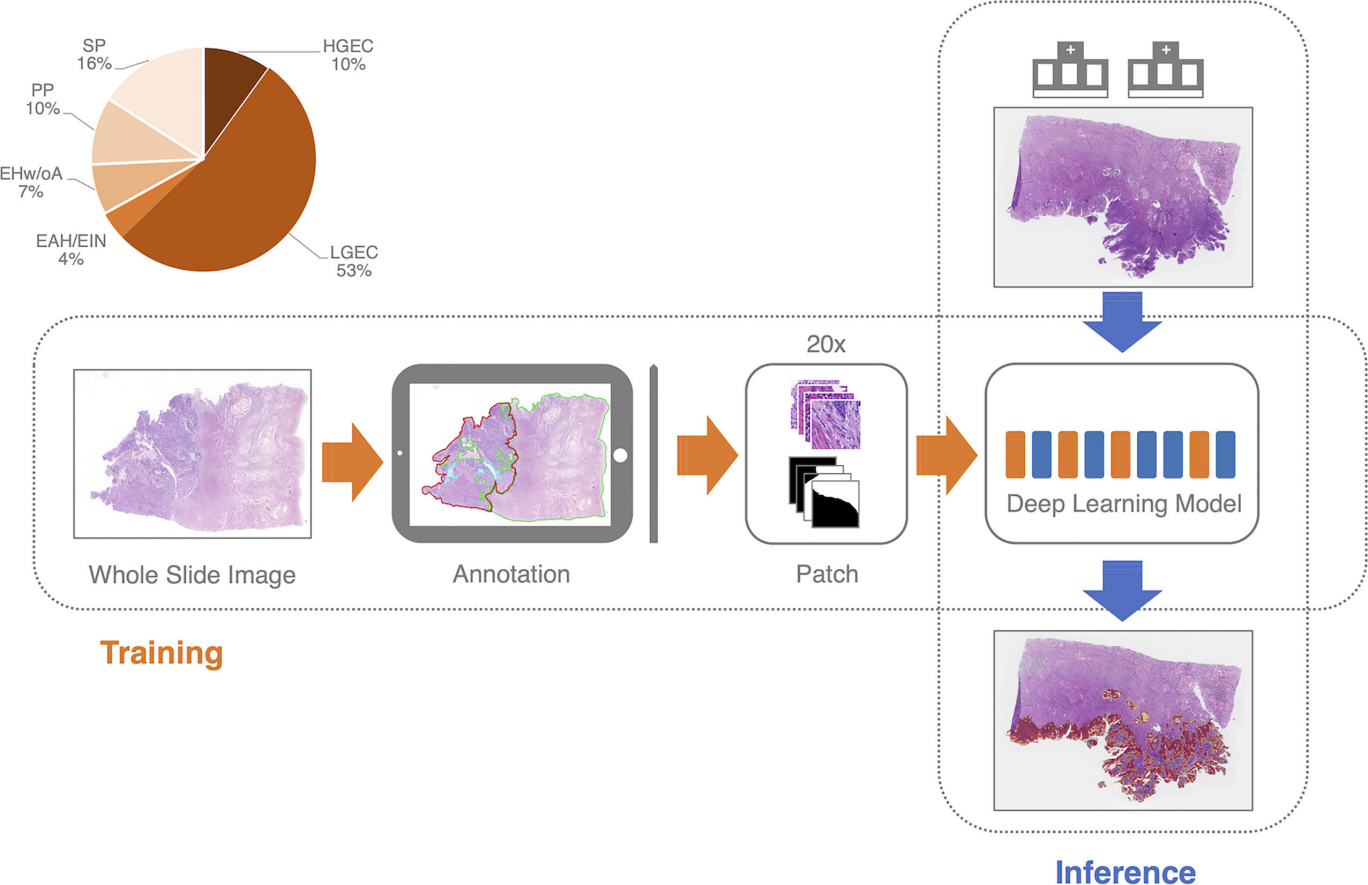
The training and inference pipeline of the deep learning model. EC, endometrial cancer; SP, Secretory phase; PP, Proliferative phase; EHw/oA, Endometrial hyperplasia without atypia; EAH/EIN, Endometrial atypical hyperplasia/Endometrioid intraepithelial neoplasia; LGEC, EC (low-grade); HGEC, EC (high-grade).

Since there are no fully connected layers, the advantage of the fully convolutional neural network design is that training and inference tile sizes do not need to be equal (**Figure 3**). During the inference phase, we divide the WSI into tiles of 2,000 by 2,000 pixels. We implemented the overlap-patch method by entering a 2,200×2,200-pixel tile into the model, but only utilized the 2,000×2,000-pixel region in the middle for the final prediction. The final prediction of the deep learning model was the EC probability for the 2,000×2,000 pixels. The tile-level predictions were then concatenated to the slide level. We used the 1000th highest pixel-level probability as the slide-level EC probability. The ROC curve was constructed from the probability by using slide-level thresholding.

### Evaluation metrics

We selected three assessment measures to characterize the model’s performance: accuracy = (TP + TN)/(TP + FN + FP + TN), sensitivity = TP/(TP + FN), and specificity = TN/(TN + FP), where TP, FP, TN, and FN stand for true positive, false positive, true negative, and false negative, respectively. Accuracy reflected the proportion of successfully predicted slides relative to the total number of slides. The sensitivity/specificity reflected the percentage of properly recognized adenomatous/normal slides. The statistics were computed using Python scripts created in-house and plotted with matplotlib.

## Results

### Model performance

**Figure 4:**
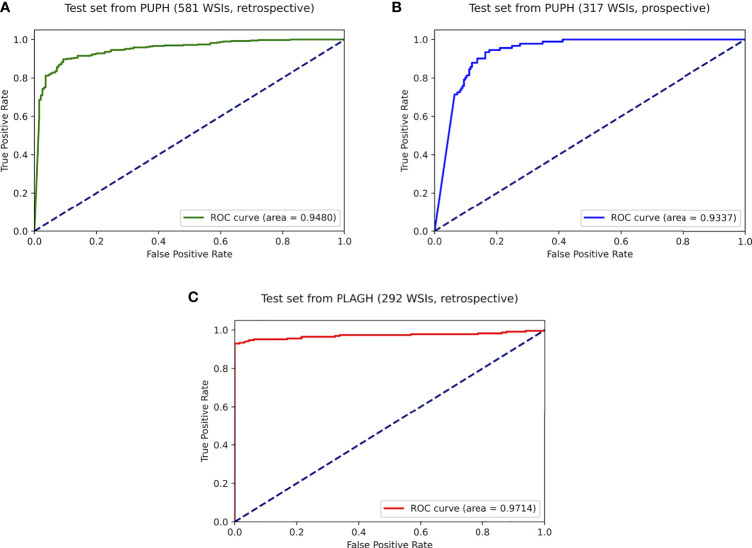
ROC curves of the best-trained model showed promising predictive power on the test datasets. **(A)** Retrospective test dataset from PUPH. **(B)** Prospective test dataset from PUPH. **(C)** Retrospective test dataset from PLAGH.

On the retrospective test set collected from PUPH, the deep learning model achieved an AUC, sensitivity, and specificity of 0.948, 0.928, and 0.800, respectively (**Figure 4A**). For the prospective study, 317 consecutive endometrium specimens for a continuous month in PUPH were collected, digitalized, and tested. The deep learning model achieved an AUC, sensitivity, and specificity of 0.933, 0.934, and 0.837, respectively (**Figure 4B**). The performance of the model on the prospective dataset was comparative to that on the retrospective one, showing the model capability on daily diagnosis.

**Figure 5:**
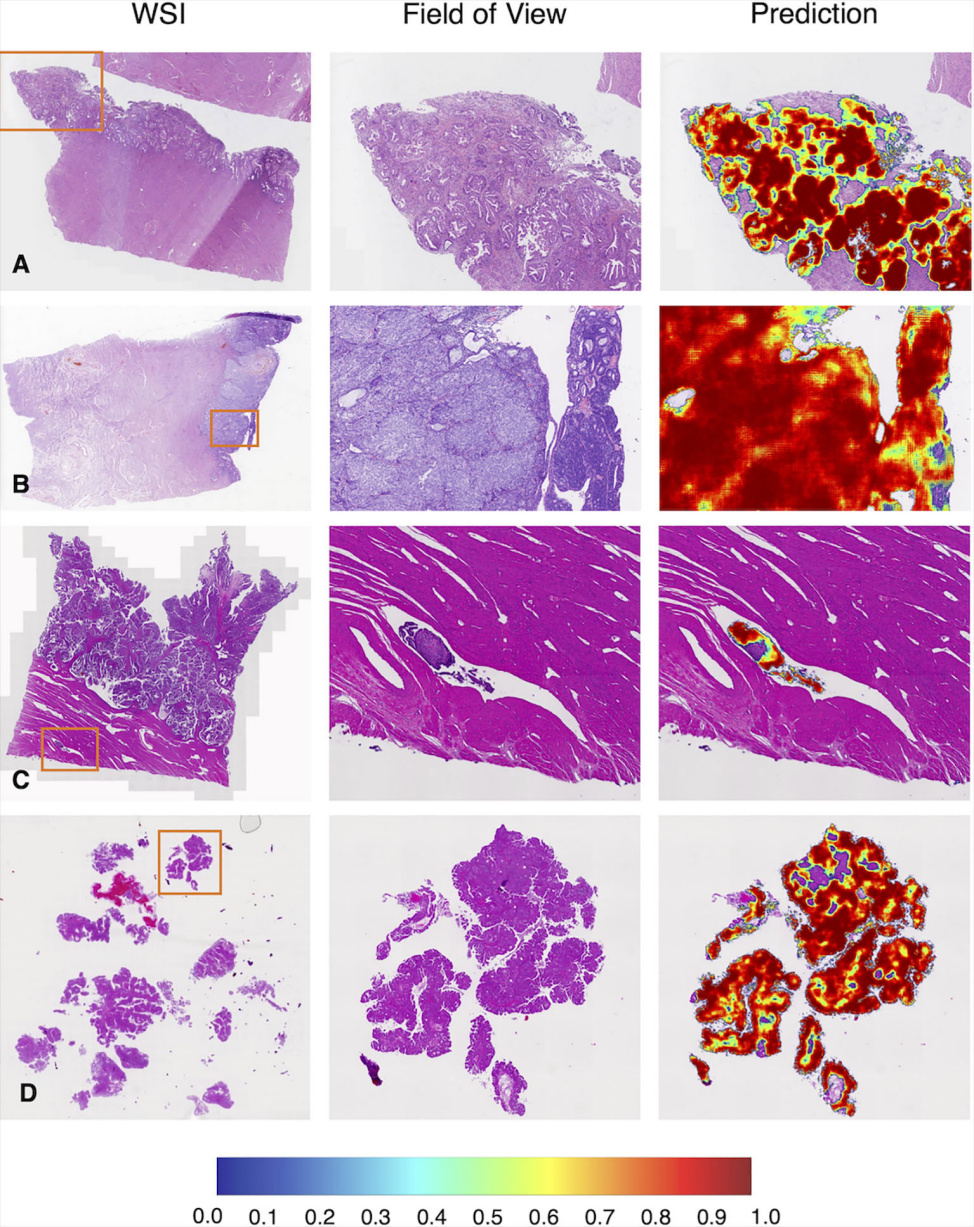
Representative examples in the test dataset from PUPH. **(A)** Low-grade EC. **(B)** High-grade EC. **(C)** Intralymphatic carcinoma thrombus. **(D)** Fragmented component of EC. WSI, whole-slide image.

As shown in **Figures 5A, B**, common subtypes of EC, including low and high-grade ECs, could be accurately detected by the deep learning model. When we focused on the predicted heatmap of the model, we found the border of the dark red area accurately fitted the cancerous regions. An example of lymph-vascular space invasion (LVSI) was given in **Figure 5C**. The model correctly identified the small infiltration lesion. A similar situation was shown in **Figure 5D**, the scattered and fragmented EC components were detected by the model.

### Multicenter study

To test the robustness of the model, 292 WSIs from PLAGH were tested. The model achieved an AUC, sensitivity, and specificity of 0.971, 0.947, and 0.938, respectively (**Figure 4C**). Despite data distribution bias, the model performance on the retrospective dataset from PLAGH was even better than that from PUPH. This finding proved the generalizability of the deep learning model.

**Figure 6:**
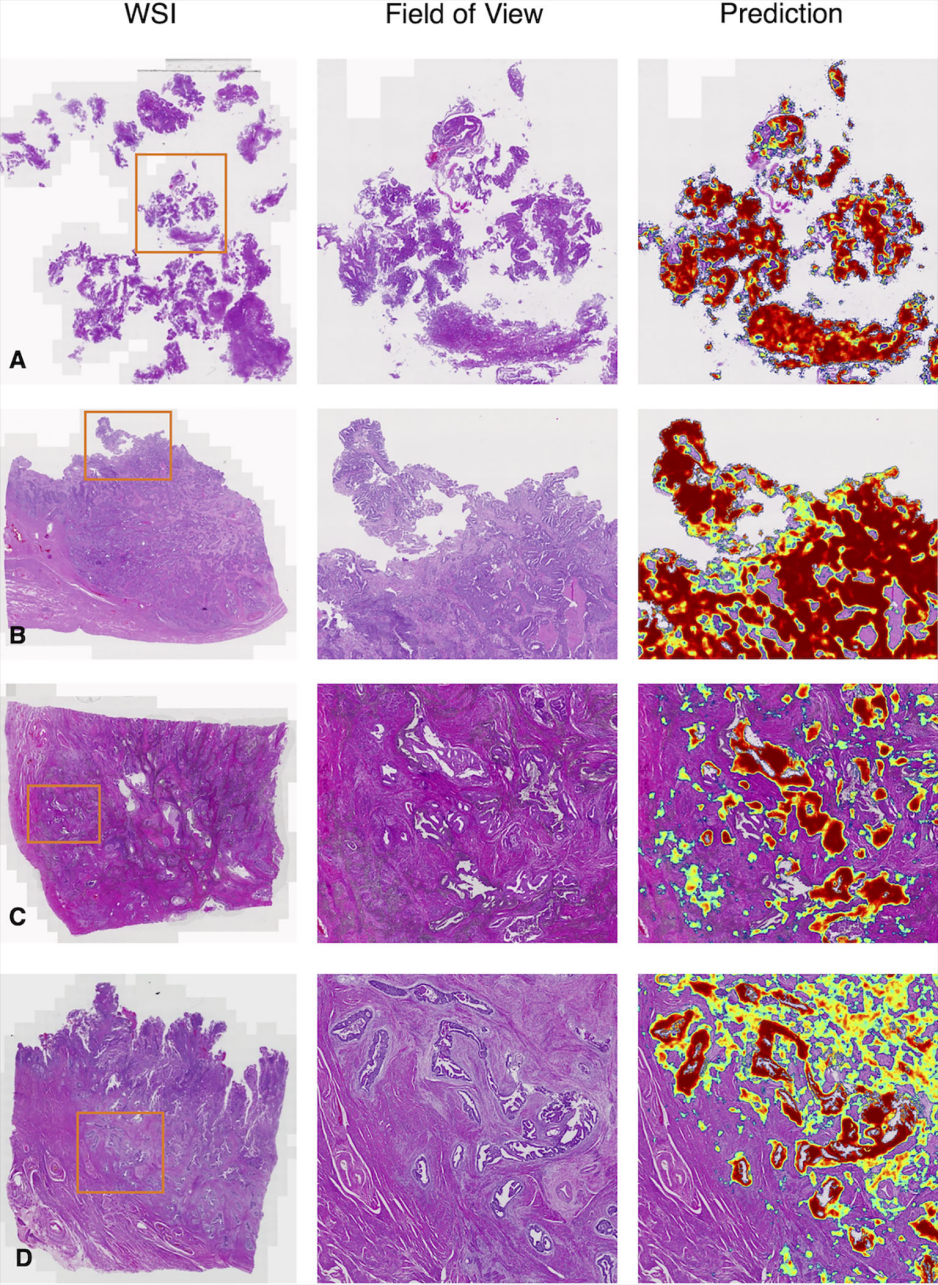
Representative examples in the test dataset from PLAGH. **(A)** Low-grade EC. **(B)** High-grade EC. **(C)** Small infiltrating lesions were showed in the deep muscularis away from the main tumor area. **(D)** MELF infiltration. WSI: whole-slide image.

We gave four predicted examples in **Figure 6**. A case of low-grade EC was shown in **Figure 6A**. The deep learning model detected cancers contained in fragmented specimens. **Figure 6B** shows a surgical specimen with high-grade EC. A more difficult situation was revealed in **Figure 6C**, with small infiltrating lesions shown in the deep muscularis away from the main tumor area. **Figure 6D** shows an example of microcystic, elongated, and fragmented (MELF) infiltration.

### False analysis

**Figure 7:**
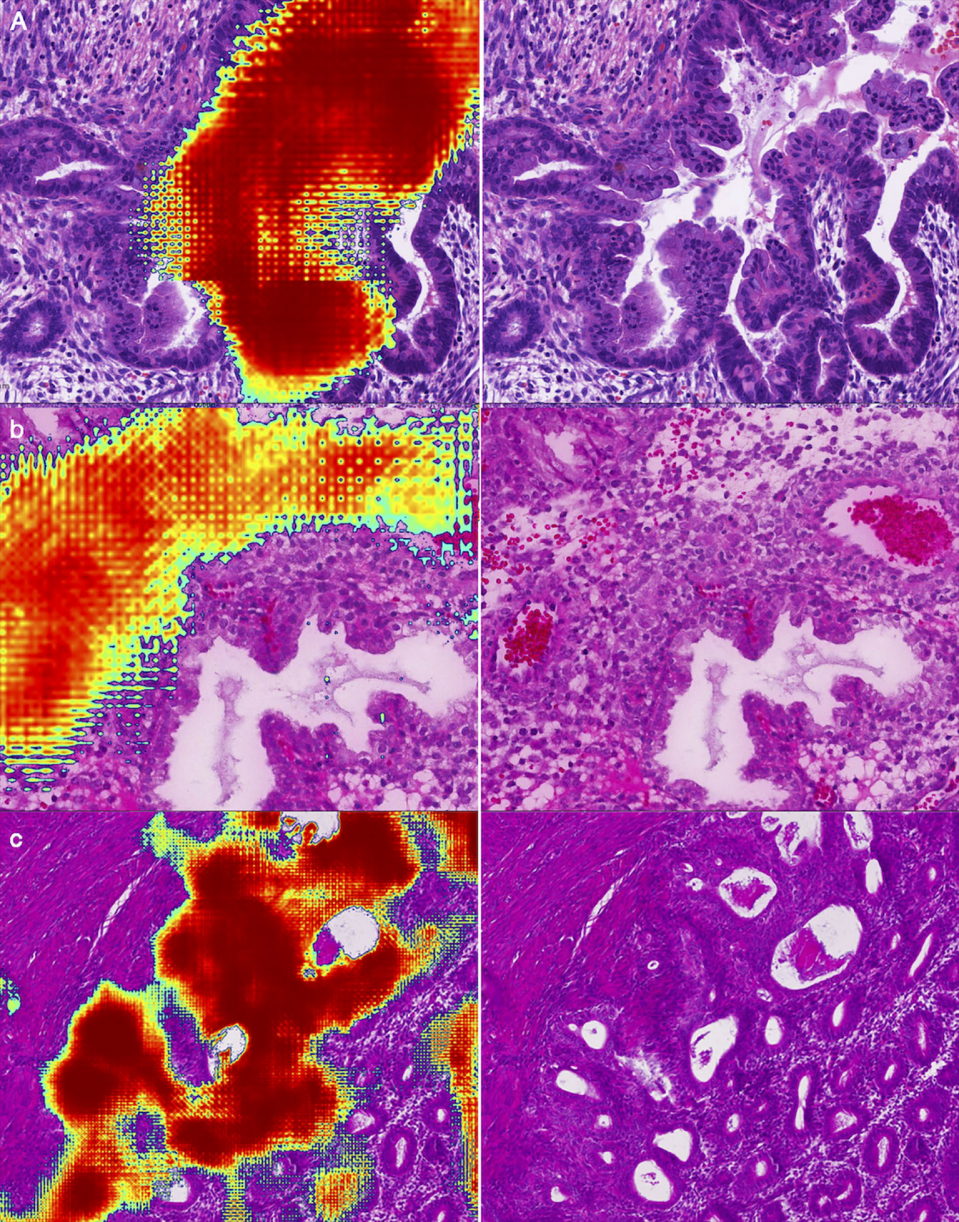
Representative examples of false positives: **(A)** Mucous metaplasia of endometrial glands with papillary hyperplasia. **(B)** Decidualization of stromal cells with epithelioid morphology. **(C)** Endometrium in the proliferative stage.

We have listed three common false positive patterns of the deep learning model in **Figure 7**. In **Figure 7A**, the mucous metaplasia of endometrial glands with papillary hyperplasia was morphologically overlapped with mucogenic EC. These lesions were cancer mimickers. It’s extremely confusing when it comes to diagnosis. In **Figure 7B**, stromal cells decidualize with epithelioid morphology. This is a typical histological feature of the secretory endometrium, similar to high grade EC in morphology. **Figure 7C** was a case of hysterectomy for multiple leiomyomas of the uterus, with endometrium in the proliferative stage. Due to the lack of submucosa in the endometrium, the interface between the endometrium and the uterine muscle wall was irregular. A few endometrial glands often appear in the superficial muscle layer, resulting in the illusion of cancer invading the uterine wall.

**Figure 8:**
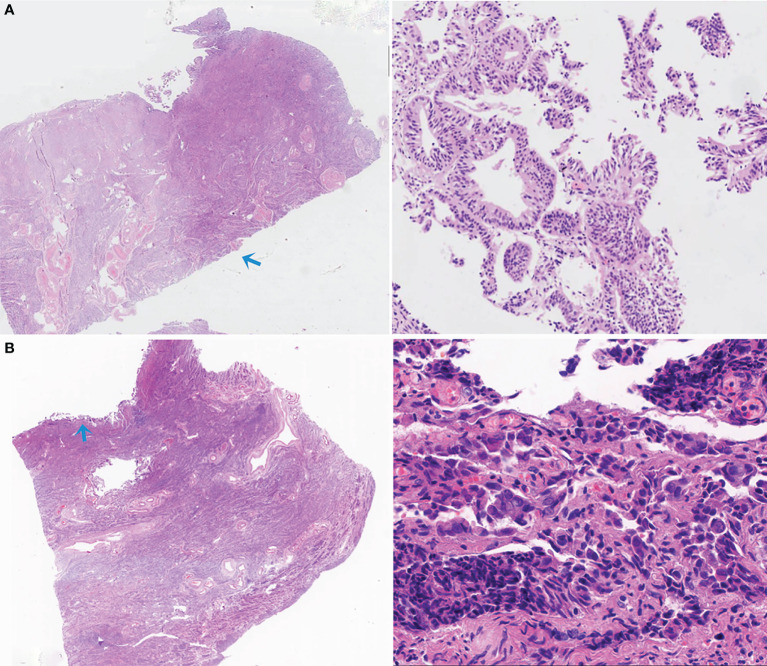
Two representative examples of false negatives. **(A)** The small, scattered, and fragmented EC components were missed by the deep leaning model. **(B)** Due to artificial factors, the compressive deformation of tissue was evident, leading to a missed diagnosis.

We are also interested in false negatives. Two representative examples of false negatives were given in **Figure 8**. In **Figure 8A**, scattered and fragmented endometrial cancer components separating from the main body of the uterus were misdiagnosed by the deep leaning algorithm. In **Figure 8B**, since the tissue was squeezed, the color of cells was dark, the nuclei were elongated, and the cell nucleoplasm ratio appeared to increase. These are similar to the morphological characteristics of cancer.

## Discussion

In recent years, artificial intelligence has achieved unprecedented development, and the application of this frontier technology in the field of medicine has gradually become a new trend. Recent studies have demonstrated promising results of deep learning algorithms in recognizing various lesions using WSIs (12, 14, 15, 21, 22). As for EC, the increasing diagnostic workload of endometrial biopsy specimens calls for the development of new models with high sensitivity and specificity.

We have developed a deep learning model to detect EC and have demonstrated the performance and generalizability of the model. Considering that the clinically significant diagnostic error rate in surgical pathology has been reported to vary from 0.26% to 1.2% (23, 24), the model performance in diagnosing EC was almost equal to that of human pathologists, suggesting that it may help pathologists under a real-world scenario as a second-opinion (25, 26).

Better diagnosis leads to better treatment for EC. LVSI is a high-risk factor for the prognosis of EC (27). The treatment approach is different for patients with or without LVSI. For human pathologists, the case shown in **Figure 5C** tends to be missed in clinical practice, especially when a pathologist is under high diagnostic pressure. The deep learning model successfully flagged these subtle regions, alerting pathologists to re-examine the case. MELF invasion was an independent prognostic factor closely related to the risk of lymph node metastasis (28), indicating poor prognosis. Omitting deep muscular infiltration leads to a lower stage (from IA2 to IA1). As shown in **Figure 6D**, the assistance of the model could help pathologists make better diagnoses.

To improve the pathologists’ confidence in the deep learning model, we performed a thorough analysis of the falsely predicted cases. In clinical practice, tissue might be significantly damaged by cauterization or compression during a biopsy, resulting in illusory appearances. These situations are also confusing for primary pathologists. A human pathologist could make a diagnosis based on a patient’s menstruation, which was not known to the model.

Most of the false positive and false negative cases in the test dataset were caused by artificial tissue deformation. These issues may be alleviated with more training data and better data augmentation techniques.

Despite all this, the deep learning model revealed excellent performance on the real-world test dataset and proved to prevent pathologists from missed diagnosis. Different from pathologists, the model is based solely on H&E-stained slides, while pathologists could review additional IHC slides and clinical data to make final diagnoses. Thus, an accurate deep learning model will not replace the breadth and contextual knowledge of pathologists. The model would function as a supplemental diagnostic tool to assist pathologists in discriminating EC from no-cancer. To boost the clinical utility value of the model, in future work, we will add more subtype identification capabilities to the model.

## Data availability statement

The original contributions presented in the study are included in the article/supplementary material. Further inquiries can be directed to the corresponding authors.

## Ethics statement

This study was approved by the Institutional Review Boards at PUPH and PLAGH. This study was conducted in accordance with the Declaration of Helsinki and informed consent from the patients was waived.

## Author contributions

XZ, SW, ZS, and DS proposed the research, XiaoyZ, XiaobZ, CW, YZ, SL, and DS performed the WSI annotation, ZS led the multicenter study, XiaobZ, WB, and SW conducted the experiment, LW, SW, and QL wrote the deep learning code and performed data analysis, XiaobZ, WB, and SW drafted the manuscript, ZS and DS reviewed the manuscript. All authors contributed to the article and approved the submitted version.

## Funding

This work is supported by the Medical Big Data and Artificial Intelligence Project of the Chinese PLA General Hospital.

## Acknowledgments

We would like to thank Cancheng Liu at Thorough Future for data collection and help discussions.

## Conflict of interest

SW is the co-founder and chief technology officer (CTO) of Thorough Future. LW is the algorithm researcher of Thorough Future.

The remaining authors declare that the research was conducted in the absence of any commercial or financial relationships that could be construed as a potential conflict of interest.

## Publisher’s note

All claims expressed in this article are solely those of the authors and do not necessarily represent those of their affiliated organizations, or those of the publisher, the editors and the reviewers. Any product that may be evaluated in this article, or claim that may be made by its manufacturer, is not guaranteed or endorsed by the publisher.
